# Effects of the direction of Kinesio taping on sensation and postural control before and after muscle fatigue in healthy athletes

**DOI:** 10.1038/s41598-023-27801-2

**Published:** 2023-01-23

**Authors:** Min-Hao Hung, Hui-Ya Chen, Yun-Chi Chang, Chun-Wen Chiu, Hsiao-Yun Chang

**Affiliations:** 1 Physical Education Office, National Chin-Yi University of Technology, Taichung, Taiwan; 2 Department of Physical Therapy, Chung Shan Medical University, Taichung, Taiwan; 3 Department of Physical Education, Fu Jen Catholic University, New Taipei City, Taiwan; 4 Institute of Athletic and Coaching Science, National Taiwan Sport University, Taoyuan, Taiwan; 5 Department of Athletic Training and Health, National Taiwan Sport University, Taoyuan, Taiwan

**Keywords:** Biophysics, Computational biophysics, Musculoskeletal system, Nervous system

## Abstract

In this study, Kinesio tape (KT) was applied in two different directions to the gastrocnemius muscle, the most important muscle in stance stability, to investigate the effect of different taping directions on overall balance and sensation systems before versus after muscle fatigue. The participants, comprising 45 healthy athletes, were randomly divided into three groups: the placebo taping group (PTG), the facilitation KT group (FKTG), and the inhibition KT group (IKTG). The tests involved in this study were a balance test, a superficial sensory function test, and a combined cortical sensation test. The data from these tests were collected before taping, after taping and a 10-min rest, and immediately after continuous heel raises were performed to fatigue. The results of the balance tests showed no significant group × time interaction, whether subjects stood barefoot on one foot or stood on a soft mat with eyes open or closed (p > 0.05). Only the sway distance and sway velocity of the center of pressure (COP) when subjects stood barefoot on one foot with eyes open were significantly higher in the inhibition taping group than in the placebo taping group (p < 0.05). In addition, significant differences were noted in the sway area and sway distance of the COP before taping, after taping, and after exercise to fatigue when the participants stood on the soft mat with their eyes open (p < 0.05). When the participants stood on the soft mat on one foot with their eyes closed, no significant differences were noted among the groups. When subjects stood on a soft mat on one foot with eyes open, significant improvements were noted after fatiguing exercise versus before taping for all three groups (p < 0.05). The results of the superficial sensory test showed no significant group × time interaction and no difference among the three taping conditions or before/after taping and after fatiguing exercise. Only in the two-point discrimination test was a sensory difference observed, with the facilitation taping group having a significantly shorter discrimination distance than the placebo taping and inhibition taping groups (p < 0.05). The present study showed that KT application for a simple balance task (e.g., barefoot on a hard floor with eyes open) may slightly influence postural control, especially when the inhibition method is used. However, more difficult balance tasks (e.g., barefoot on a soft mat with eyes closed) show no effect of KT application—either the facilitation method or the inhibition method—on posture control.

## Introduction

Most sports injuries occur in the middle and late stages of competition or practice and are correlated to some extent with fatigue^1,2^. Fatigue reduces an athlete’s neuromuscular control^3^, thus decreasing balance. With muscle fatigue, the control of joint positioning becomes less precise.

Balance is an essential ability for athletes. Techniques in sports such as gymnastics, ball games, and track and field are particularly demanding with respect to balance, making it essential for athletic performance^4^. Most people rely on dynamic or static balance while standing, walking, or running. Static balance maintains a stable posture, whereas dynamic balance enhances the quality of movement^5^. Balance is controlled by four factors within two systems in the human body: the factors of vision, vestibular function, and proprioception in the nervous system and the factor of muscle strength in the musculoskeletal system^6^. Balance is achieved by adjusting the relative positions of the center of gravity and base of support to achieve stability. The body must continuously correct and adjust the angles of the ankle and hip joints to maintain static balance while standing. The ankle joint muscles are used primarily in the forward and backward directions, whereas the hip joint muscles are mainly responsible for left–right adjustments. The information sources for these adjustments rely on the continuous input of proprioceptive signals^7^.

Regarding static balance, standing is the most functional stance for postural assessment. The ability to control the ankle joint while standing is an essential factor affecting standing balance^8^. The standing static balance test can be used to determine the sensory and muscle coordination of the test subject^9^. The gastrocnemius muscle is the most important stabilizing muscle for standing. Previous studies have suggested that subjects with poor proprioception have poor stance quality, which increases their risk of falls and reduces their ability to carry out activities of daily living^10^. In a clinical setting, constant stimulation is used to compensate for and enhance the sources of proprioception.

Kinesio tape (KT) is a widely available auxiliary device with a proven ability to enhance proprioceptive input in many studies^11^. KT was experimentally shown to stimulate tactile input when applied to the skin; affect mechanoreceptors; enhance proprioception in the joints, muscles, and tendons^11,12^; improve myoelectric activity and recruitment^13–16^; and improve ankle joint position sense in healthy individuals and individuals with sports injuries^17^.

Further studies have found that KT can improve proprioception in subjects^17–19^ and enhance dynamic balance in athletes^20–22^. Different taping methods can result in different effects; accordingly, users have employed different KT shapes, tensions, and directions as needed^23^. Taping methods can be classified based on direction. In the facilitation method, the goal is to assist muscle contraction, whereas in the inhibition method, the goal is to relax the muscles and increase flexibility or joint mobility. Some studies suggest that facilitation taping can increase muscle strength, power, recruitment, and tension^24,25^, but some researchers believe that facilitation and inhibition methods do not affect muscle contraction or coordination^18,26–28^. Facilitation methods produce an eccentric pull on the underlying fascia, inhibiting or decreasing muscle contraction^27^.

However, previous studies focused primarily on the coordination of a single muscle and did not test the overall contraction performance and coordination of the muscle group simultaneously. In addition, opinions differ about facilitation and inhibition taping methods^18,24–28^.

Although KT has been demonstrated to improve proprioception, such as joint position sense and force sense^17^. The human somatosensory system can be divided into three categories: superficial, deep, and combined cortical. Superficial sensation, such as light touch, is produced by stimulating the skin and subcutaneous tissues^29–32^; deep sensation, such as vibratory sense, joint position sense, and force sense, is produced by stimulating muscles, tendons, ligaments, joints, and fascia; and combined cortical sensation, such as two-point discrimination, is produced by the combination of superficial and deep sensations^11,17,21,30^. KT may produce interactions among these three sensation systems, thus influencing proprioception. Current studies suggest that KT can improve deep sensation (joint position sense and force sense)^17,21,33^, but many researchers hold the opposite view^28,34,35^; moreover, the effects of other sensations have not been sufficiently studied. Therefore, in this study, KT was applied in two different directions to the gastrocnemius muscle, the most important muscle in stance stability, to further investigate the effect of different taping directions on overall balance and sensory systems before versus after muscle fatigue.

It was hypothesized that the facilitation taping group would perform better on balance and sensory tests than the other two groups. Because of the role of the gastrocnemius muscle in balance and stability, KT interventions involving different taping directions were used for the gastrocnemius muscle to determine whether the direction of KT application can produce significant benefits in terms of the function, sensation, and performance of the gastrocnemius muscle.

## Results

The results of the balance tests are shown in Tables 1, 2, 3 and 4. No significant group × time interaction was found, whether subjects were standing barefoot on one foot or standing on a soft mat with eyes open or closed eyes condition (*p* > 0.05). Only the COP sway distance and sway velocity in subjects standing barefoot on one foot in the eyes-open condition were significant higher in the inhibition taping group than in the placebo taping group (*p* < 0.05). In addition, significant differences were noted in the COP sway area and sway distance before taping, after taping, and after fatiguing exercise when the participants stood on the soft mat with their eyes open (*p* < 0.05).

**Table 1:** The balance data of three taping groups on barefoot with eyes open and eyes closed condition.

	Placebo taping			Facilitation taping			Inhibition taping		
	Before	After	Fatigue	Before	After	Fatigue	Before	After	Fatigue
Eyes open									
Standing time (s)	10.0 ± 0.1	10.0 ± 0.0	10.0 ± 0.0	10.0 ± 0.0	10.0 ± 0.1	9.4 ± 2.3	10.0 ± 0.0	10.0 ± 0.1	9.9 ± 0.6
COP area (mm^2^)	182.99 ± 111.47	200.96 ± 173.48	324.33 ± 411.65	213.21 ± 208.51	308.73 ± 248.54	211.23 ± 106.81	279.51 ± 271.88	278.89 ± 252.48	1070.79 ± 3500.37
COP displacement (mm)^**§**^	226.12 ± 42.75	218.87 ± 69.64	249.31 ± 74.26	250.48 ± 64.59	295.89 ± 66.53	247.32 ± 57.18	311.43 ± 108.92	330.49 ± 112.99	313.38 ± 166.12
COP velocity (mm/s)^$^^,^^&^	22.89 ± 4.32	24.17 ± 3.82	25.21 ± 7.31	25.39 ± 6.54	29.96 ± 6.79	25.04 ± 5.82	31.67 ± 11.04	33.53 ± 11.39	41.51 ± 36.64
Eyes closed									
Standing time (s)	10.0 ± 0.1	9.72 ± 0.84	9.57 ± 1.74	8.81 ± 2.48	9.56 ± 1.79	9.86 ± 0.73	8.58 ± 2.74	9.01 ± 2.24	8.69 ± 2.65
COP area (mm^2^)	692.29 ± 584.36	700.53 ± 585.31	650.63 ± 390.58	731.06 ± 566.03	782.78 ± 479.03	809.31 ± 606.70	935.22 ± 1056.88	653.67 ± 545.81	1473.13 ± 2277.59
COP displacement (mm)	592.38 ± 166.55	494.21 ± 186.89	482.78 ± 112.61	498.25 ± 218.34	615.33 ± 193.77	555.00 ± 163.52	459.51 ± 164.90	520.15 ± 221.07	576.90 ± 279.04
COP velocity (mm/s)	59.96 ± 16.91	51.56 ± 18.37	53.58 ± 19.90	117.51 ± 241.36	63.89 ± 15.99	57.43 ± 17.24	59.11 ± 22.08	100.12 ± 149.69	74.45 ± 46.30

^**§**^Significant difference between Inhibition taping group and Placebo taping group in before, immediate after taping and after fatigue exercise.

^$^Significant difference between Inhibition taping group and Placebo taping group in before, immediate after taping and after fatigue exercise.

^**&**^Significant difference between Inhibition taping group and Facilitation taping group in before, immediate after taping and after fatigue exercise.

**Table 2:** Statistical results of the balance data among the three groups on barefoot with eyes open and closed eyes condition.

	Within-subject (pre-post-post fatigue)			Between-subject (group)			Interaction (group × time intervention)			
	F(2,84)	P value	Partial eta squared	F(1,42)	P value	Partial eta squared	F(2,84)	P value	Partial eta squared	Power
Eyes open										
Standing time (s)	1.502	0.229	0.035	0.750	0.479	0.034	0.749	0.561	0.034	0.232
COP area (mm^2^)	1.073	0.347	0.025	0.750	0.479	0.034	0.823	0.514	0.038	0.253
COP displacement (mm)	0.920	0.402	0.021	5.300	0.009*	0.202	1.364	0.254	0.061	0.408
COP velocity (mm/s)	1.002	0.371	0.023	6.687	0.003*	0.242	1.012	0.406	0.046	0.307
Eyes closed										
Standing time (s)	0.378	0.686	0.009	2.046	0.142	0.089	0.825	0.513	0.038	0.253
COP area (mm^2^)	1.471	0.239	0.034	0.845	0.437	0.039	1.560	0.192	0.069	0.463
COP displacement (mm)	0.273	0.762	0.006	0.390	0.680	0.018	2.270	0.068	0.098	0.640
COP velocity (mm/s)	0.371	0.691	0.009	0.807	0.453	0.037	1.110	0.357	0.050	0.335

* *p* < .05.

**Table 3:** The balance data of three taping groups on soft-mat with eyes open and eyes closed condition.

	Placebo taping			Facilitation taping			Inhibition taping		
	Before	After	Fatigue	Before	After	Fatigue	Before	After	Fatigue
Eyes open									
Standing time (s)	10.02 ± 0.09	10.04 ± 0.08	9.81 ± 0.76	9.49 ± 1.83	9.67 ± 1.32	9.99 ± 0.12	9.88 ± 0.52	10.00 ± 0.03	10.00 ± 0.08
COP area (mm^2^)^£^	513.33 ± 547.74	349.65 ± 252.24	355.33 ± 349.66	663.21 ± 619.43	406.19 ± 206.87	512.33 ± 354.36	674.49 ± 621.13	464.25 ± 513.21	474.03 ± 492.35
COP displacement (mm)^¶^	275.29 ± 131.76	271.85 ± 62.81	258.05 ± 71.27	340.15 ± 116.94	299.76 ± 85.34	307.46 ± 53.95	422.75 ± 295.34	305.95 ± 109.26	309.42 ± 116.47
COP velocity (mm/s)	30.85 ± 8.90	27.45 ± 6.20	26.70 ± 6.92	59.92 ± 89.06	31.91 ± 7.97	31.19 ± 5.43	45.30 ± 38.46	31.05 ± 10.86	31.53 ± 11.88
Eyes closed									
Standing time (s)	4.75 ± 2.22	5.49 ± 2.73	5.94 ± 2.44	5.11 ± 2.17	4.99 ± 2.64	6.24 ± 3.43	4.58 ± 2.21	4.33 ± 2.21	5.72 ± 2.65
COP area (mm^2^)	2114.84 ± 2117.01	2230.77 ± 1922.22	2456.55 ± 3416.70	2275.95 ± 1502.05	1860.61 ± 1467.55	2080.97 ± 1796.78	4254.07 ± 9600.39	1582.62 ± 1536.49	1394.31 ± 661.20
COP displacement (mm)	397.96 ± 186.08	448.93 ± 230.62	468.08 ± 239.81	473.69 ± 221.18	404.33 ± 226.02	486.43 ± 287.81	423.15 ± 183.69	341.48 ± 278.82	456.55 ± 169.74
COP velocity (mm/s)	86.97 ± 23.14	84.77 ± 27.50	82.34 ± 24.00	93.61 ± 22.56	82.92 ± 21.94	140.17 ± 233.43	98.31 ± 35.59	76.06 ± 24.00	86.85 ± 31.61

^£^Significant difference between before and after taping, and between before and after fatigue exercise for three group.

^¶^Significant difference between before and after fatigue exercise for three group.

**Table 4:** Statistical results of the balance data among the three groups on soft-mat with eyes open and eyes closed condition.

	Within-subject (pre-post-post fatigue)			Between-subject (group)			Interaction (group × time intervention)			
	F(2,84)	P value	Partial eta squared	F(1,42)	P value	Partial eta squared	F(2,84)	P value	Partial eta squared	Power
Eyes open										
Standing time (s)	0.351	0.705	0.008	1.351	0.270	0.60	0.767	0.550	0.035	0.237
COP area (mm^2^)	7.236	0.001*	0.147	0.454	0.638	0.021	0.198	0.939	0.009	0.090
COP displacement (mm)	4.162	0.019*	0.090	2.068	0.139	0.090	1.460	0.222	0.065	0.435
COP velocity (mm/s)	3.359	0.073	0.074	1.533	0.228	0.068	0.724	0.578	0.033	0.225
Eyes closed										
Standing time (s)	4.001	0.059	0.087	1.031	0.366	0.047	0.317	0.866	0.015	0.118
COP area (mm^2^)	1.310	0.275	0.030	0.064	0.938	0.003	1.254	0.295	0.056	0.377
COP displacement (mm)	1.354	0.264	0.031	0.381	0.686	0.018	0.496	0.739	0.023	0.163
COP velocity (mm/s)	0.867	0.424	0.020	0.770	0.469	0.035	0.840	0.503	0.038	0.258

*p < 0.05.

When the participants stood on the soft mat on one foot with their eyes closed, no significant differences were noted among the groups. When they stood on a soft mat on one foot with eyes open, all three groups showed significant better results after fatiguing exercise than before taping (*p* < 0.05).

The results of the superficial sensory test showed no significant group × time interaction and no difference among the three taping conditions or before/after taping and after fatiguing exercise (Tables 5 and 6). Only in the two-point discrimination test was a sensory difference observed, with the facilitation taping group having a significantly shorter discrimination distance than the placebo taping and inhibition taping groups (*p* < 0.05).

**Table 5:** Superficial sensory test results of three taping groups.

Placebo taping			Facilitation taping			Inhibition taping		
Before	After	Fatigue	Before	After	Fatigue	Before	After	Fatigue
Vibratory sense (s)								
31.72 ± 6.88	32.80 ± 8.06	31.48 ± 6.92	31.30 ± 5.85	29.47 ± 3.63	28.78 ± 4.8	29.22 ± 8.03	30.27 ± 8.55	32.01 ± 9.07
Two-point discrimination (cm)^¥^								
7.47 ± 2.16	7.10 ± 1.50	7.30 ± 2.27	5.57 ± 2.22	5.83 ± 2.12	5.57 ± 2.43	7.30 ± 2.27	7.00 ± 1.61	7.00 ± 1.40

^¥^Significant difference between facilitation taping and Placebo taping; between facilitation taping and Inhibition taping.

**Table 6:** Statistical results of superficial sensory test among the three groups of placebo taping, inhibition taping, and facilitation taping.

	Within-subject (pre-post-post fatigue)			Between-subject (group)			Interaction (group × time intervention)			
	F(2,84)	P value	Partial eta squared	F(1,42)	P value	Partial eta squared	F(2,84)	P value	Partial eta squared	Power
Vibratory sense	0.007	0.993	0.000	0.494	0.614	0.023	1.586	0.186	0.070	0.470
Two-point sensory	0.364	0.696	0.009	3.992	0.026*	0.160	0.355	0.840	0.017	0.127

*p < 0.05.

## Discussion

### Sensation

The results of this study showed that Kinesio taping had no effect on vibratory sense but had significant benefits for two-point discrimination after taping, specifically with the facilitation taping method. Most previous studies focused on deep sensation position and strength before versus after taping^11,17^, but some researchers have posited that KT has no effect on position sense or force sense^28,34,35^. The present study also found that KT had no effect on vibratory sense, which is classified as a deep sensation, but it did affect two-point discrimination, which is classified as combined cortical sensation. Furthermore, the present study found that facilitation taping improved two-point discrimination and increased the sensitivity of combined cortical sensation. This indicates that facilitation taping, which is consistent with the direction of muscle contraction, can enhance skin sensation. In a previous study, facilitation and inhibition taping were used on the outer edge of the forearm of ten healthy individuals who were asked to flex and extend the wrist repeatedly. The results of sensorimotor coordination tests showed that KT improved the subjects’ muscle coordination^19^. Our study supported this previous study by producing similar results. KT taping with facilitation methods may improve muscle coordination by increasing combined cortical sensation. However, our study did not measure position sense or touch sense to better understand the effect of KT on sensation. This is one of the limitations of our study.

### Balance

The present study revealed no significant differences among groups when the participants stood barefoot on one foot with eyes closed. However, when they stood barefoot on one foot with eyes open, the sway distance of the inhibition taping group was significantly greater than that of the placebo taping group before taping, after taping, and after fatiguing exercise, and the sway velocity was significantly higher in the inhibition taping group than in the placebo group or the facilitation taping group. It has been reported previously that KT with inhibition methods loosens the adhesion between the skin and the underlying tissue^18,19,23^. This may result in an increase in the space between the skin and the subcutaneous tissue as well as a decrease in sensory stimulation and muscle coordination^18,19,23^. Hence, the sway velocity and sway distance of the COP increased.

When participants stood on a soft mat on one foot with their eyes open, the sway area and sway distance of the COP in all three groups gradually decreased before taping, after taping, and after fatiguing exercise. These results indicated that cutaneous blood flow may increase following fatiguing exercise, which would improve muscle metabolism and enhance mechanoreceptor stimulation. This mechanism may cause the sway area and sway distance of the COP to decrease after fatiguing exercise^36^. However, there was no difference among the three taping groups when participants stood on a soft mat on one foot with eyes open. In addition, there was no significant improvement in balance among the three taping groups before taping, after taping or after fatiguing exercise. During static balance, a lack of visual feedback increases the difficulty of maintaining balance because proprioceptive input is the only remaining source of feedback. This may be because the tests were conducted immediately after taping, when coordination between proprioception and neuromuscular control has not yet been achieved because it does not occur immediately. In addition, a lack of visual feedback and the disturbance of proprioception by a soft mat enhance the difficulty of maintaining balance, so that even if KT stimulates cutaneous mechanoreceptors, it is not sufficient for the participants to maintain balance.

When standing still, the human body adjusts the position of its center of mass over the base of support to maintain balance^37^. In the present study, the extent and velocity of COP movement were measured to evaluate the subject’s stability in terms of swaying, proprioception, and posture^38–42^. In the present study, the gastrocnemius was taped because forward–backward displacement of the center of pressure when standing still is affected by the use of the ankle strategy for posture control^7^, and the contractile state of the gastrocnemius directly affects ankle control ability. Previous studies reported that KT intervention before exercise can improve dynamic balance in healthy athletes^20–22^, but these studies did not involve intervention after fatiguing exercise. The present study included intervention after fatiguing exercise and divided the taping methods into facilitation and inhibition taping to elucidate the effects of these two methods. In a study where the gastrocnemius was taped in different directions, the taping direction did not increase vertical jumping ability but changed the electromyographic activation state of the gastrocnemius^14^. Previous studies on different directions of taping showed that facilitation taping of the quadriceps increased torque in the knee joint^25^. The findings of the present study are not consistent with those of previous studies.

Prolonged exercise causes muscle fatigue and affects balance control^6^, and muscle fatigue causes pain and decreased motor control^43,44^. Fatigue of the gastrocnemius reduces ankle joint stability^3^. Preventing fatigue of the gastrocnemius is necessary for accurate control of the ankle joint^45^. Previous studies reported that KT effectively reduces pain immediately^46^ and that it also diminishes muscle fatigue^47^ and the incidence of ankle sprains^47,48^. Furthermore, the present study further showed that gastrocnemius KT cannot improve balance control after fatiguing exercise. This contrasts with the results of previous studies showing that KT before fatigue intervention can reduce the tendency for balance to decline after fatigue^49^.

This research had some limitations. In contrast to previous studies that used COP parameters to evaluate static balance as subjects stood on one leg for 10 s, the participants in our study could not complete the difficult balance task with their eyes closed. Therefore, we recorded the time until participants lost their balance in a standing task and we used that parameter in our study, which was also a limitation in our study.

In conclusion, the findings in the present study showed that balance maintenance in a simple task (e.g., barefoot on hard floor and eye open) may have little influence on postural control, especially in the inhibition method of the KT applied. However, more difficulty balance tasks (e.g., barefoot on soft mats and eyes closed) would have no effect of KT applied, whether facilitation or inhibition methods, on posture control.

## Methods

### Experimental approach to the problem

In this study, two different taping directions were applied to the gastrocnemius muscle, the most important muscle for stance stability, to further investigate the effect of different taping directions on overall balance and sensation systems before versus after muscle fatigue. Forty-five healthy athletes were recruited for this study (Table 7). The inclusion criteria for healthy athletes were as follows: (1) the participants reported no history of surgery on the lower limbs or musculoskeletal disorders in a span of 1 year prior to data collection; (2) the frequency of sports training was more than 3 days per week, and the participants had at least three years of training experience in their sport; and (3) the participants had never had a medical problem that affected balance, such as head injury, concussion, otitis media, Meniere disease, or hearing loss. Approval from the relevant local Institutional Review Board (Cheng Ching Hospital Institutional Review Board [07/05/2017; IRB No: HP170013]) and individual written informed consent from all participants were obtained beforehand. All experiments were performed in accordance with relevant local guidelines and regulations.

**Table 7:** Mean ± SD of demographic characteristics of participants.

Variable	PTG	FKTG	IKTG	F	*P*
Number of participants (male: female)	15 (6:9)	15(12:2)	15(7:8)	–	–
Age (years)	20.5 ± 1.7	19.9 ± 1.4	19.8 ± 1.0	1.186	.316
Height (cm)	165.9 ± 9.3	172.0 ± 7.9	166.7 ± 9.4	2.085	.137
Body mass(kg)	61.1 ± 13.0	64.3 ± 8.6	63.3 ± 14.1	.282	.755
Training frequency (time/week)	3.5 ± 1.5	4.4 ± 1.5	3.4 ± 1.8	1.744	.187
Training time (hour/week)	2.2 ± 1.0	2.8 ± 1.2	2.5 ± 1.0	1.109	.339

### Experimental design

The participants were randomly divided into three groups: the placebo taping group (PTG), the facilitation KT group (FKTG), and the inhibition KT group (IKTG). The tests involved in this study were the balance test, superficial sensory function test, and combined cortical sensation test. Data were collected before taping, after taping and a 10-min rest, and immediately after continuous heel raises were performed to the point of fatigue.

### Procedures

#### Application direction of Kinesio tape

**Figure 1:**
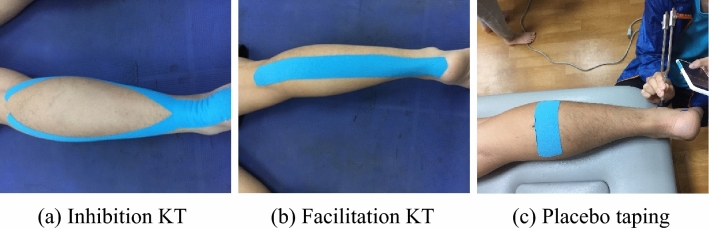
Applied direction of Kinesio taping (**a**) inhibition KT, (**b**) facilitation KT, (**c**) placebo taping.

A two-inch (5 cm) Kinesio tape (Kinesio Tex Tape, Kinesio Holding Company, Albuquerque, NM) was used in this study, and the stretch tension of the tape was 100%, which means that we stretched the length of the tape from the bottom layer of the self-adhesive paper tear to 140% of the original length of the tape. The taping site was the gastrocnemius muscle, and the taping methods were placebo taping, inhibition taping, and facilitation taping (Fig. 1).

##### Inhibition taping

Tape was applied from the end (at the bottom of the heel) to the beginning of the muscle (at the back of the knee) while the knee was extended and the foot was pushed down into a dorsiflexed position. A 10-cm piece of tape was applied to the sole of the foot, a Y-shaped strip was applied along the outer edge of the calf, and another strip of tape was applied along the inner edge of the calf. The tension of the tape was 100% when the knee was passively straightened (Fig. 1a).

##### Facilitation taping

Tape was applied from the beginning (at the back of the knee) to the end of the muscle (at the bottom of the heel). The tape was placed in an I-shape while the subject's knee joint was extended and the ankle was in a plantar flexion position. The tape was applied from the back of the knee to the heel, and the tension of the tape was 100% (Fig. 1b).

##### Placebo taping

On the midsection of the gastrocnemius, an I-shaped horizontal strip was attached at 100% tension (Fig. 1c).

### Fatiguing exercise

Gastrocnemius muscle fatigue is determined by continuous heel raising movement and defined as the point when the height of the heel is less than half of the starting point three consecutive times^49^.

### Testing procedures

**Figure 2:**
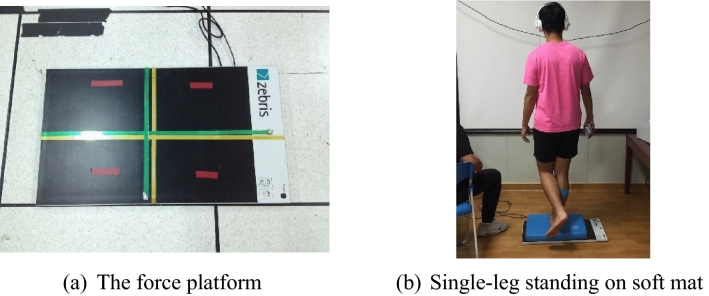
(**a**) The force platform and (**b**) test for single-leg standing on soft mat condition.

A force plate (Zebris FDM-S, Zebris Medical GmbH, Germany) was used in this study. The sampling frequency of the Zebris force platform was set at 100 Hz. The change in center of pressure (COP) shifting was used to evaluate the neuromuscular balance ability of the lower extremities. This study was conducted in a single-leg stance, with the dominant leg as the supportive leg. The dominant leg was defined as the leg that steps up first when climbing stairs and the leg that is used to kick a ball. There were four test conditions in this study: (1) open-eye, barefoot, single-leg standing; (2) closed-eye, barefoot, single-leg standing; (3) open-eye, single-leg standing on a soft mat; and (4) closed-eye, single-leg standing on a soft mat. The measurement variables included time spent standing on one leg, area of body sway, path length of body sway, and velocity of body sway (Fig. 2).

### Superficial sensory function test

**Figure 3:**
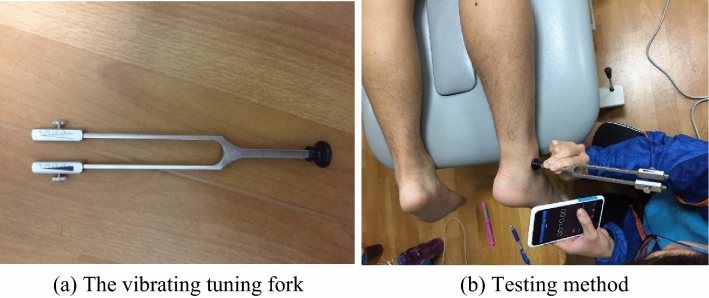
Device for superficial sensory test (**a**) the vibrating tuning fork; (**b**) testing method.

A vibrating tuning fork (Fig. 3a) and a two-point discrimination tool (Two-Point Aesthesiometer, Lafayette Instrument, Lafayette, IN) were used to conduct superficial sensory tests (Fig. 3a). The vibratory sense was tested using a 128 Hz vibrating tuning fork. During the test, subjects were required to close their eyes and wear headphones; the tuning fork was first struck and then placed on the lateral ankle bone to let the subjects feel its vibration of the tuning fork, and the timer was started. When the subject no longer felt the vibration of the tuning fork, the timer was stopped, and the value on the tuning fork and the stop time were recorded (Fig. 3b). Three measurements were taken during the test, and the average of these three measurements was used as the final value.

### Combined cortical sensation test

**Figure 4:**
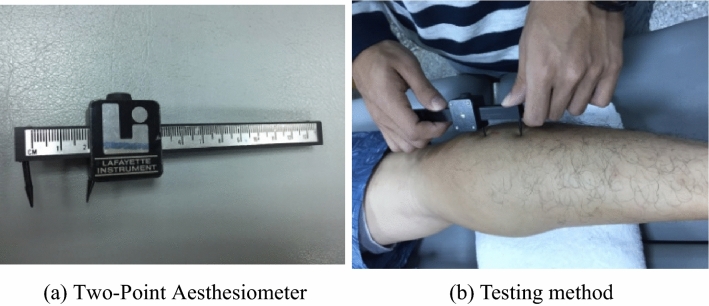
Device for combined cortical sensation test (**a**) the two-point aesthesiometer; (**b**) testing method.

The distance between the two ends of the two-point discrimination tool (Fig. 4a) started at a width of 10 cm and was adjusted down by 1 cm at each step. The subject lay on the bed for each test, and the distance in centimeters was measured until the subject could not distinguish the two ends of the tool; the length of the last distinguishable distance was recorded (Fig. 4b).

### Statistical analyses

The detection time points were before taping, immediately after taping, and after continuous heel raising movement performed to fatigue. To examine the differences among the comparisons, repeated-measures, mixed-design, two-way ANOVA (group*time intervention) was conducted in IBM SPSS Package Software 22.0. If group-by-time interactions were significant, LSD-adjusted post hoc tests were computed. The three situation parameters were before, after taping, and after fatigue from heel-raise exercise; the taping groups were the placebo taping group (PTG), facilitation KT group (FKTG), and inhibition KT group (IKTG). The significance level was set to *a* = 0.05.

## Data Availability

The datasets generated and/or analyzed during the current study are available from the corresponding author on reasonable request.
